# The Influence of Disease Status on Loneliness of the Elderly: Evidence from Rural China

**DOI:** 10.3390/ijerph19053023

**Published:** 2022-03-04

**Authors:** Jiahao Song, Haitao Wu, Hongxing Lan, Dingde Xu, Wei Wang

**Affiliations:** 1 Department of Rural and Regional Development, College of Management, Sichuan Agricultural University, 211 Huimin Rd, Chengdu 130062, China; songjiahao@sicau.edu.cn; 2 Department of Agricultural Economics and Management, School of Business Administration, Zhongnan University of Economics and Law, 182 Nanhu Rd, Wuhan 430073, China; wuhan_haitao@aliyun.com; 3 Sichuan Center for Rural Development Research, College of Management, Sichuan Agricultural University, 211 Huimin Rd, Chengdu 130062, China; 13504@sicau.edu.cn; 4 Department of Agriculture and Forestry Economics and Management, College of Management, Sichuan Agricultural University, 211 Huimin Rd, Chengdu 130062, China

**Keywords:** rural elderly, diseases, aloneness, serious illness

## Abstract

At present, the dual pressure of rural labor outflow and population aging in China makes the problems of the rural elderly population increasingly prominent, and its health problem is particularly prominent. Based on the 2014 China elderly population health survey data (CLHLS), this paper finds that the physical health status of the rural elderly has a significant positive impact on their loneliness; that is, the rural elderly with poor health status are more likely to feel lonely. At the same time, the age of the elderly has a significant positive impact on their loneliness. On the contrary, gender, personality, family income and intergenerational support of the elderly have a negative impact on their loneliness. Chronic diseases such as hypertension and diabetes have no significant effect on the loneliness of the elderly in rural areas, but there is a “severe disease effect”; that is, when chronic diseases develop into serious diseases or acute serious diseases, it can negatively impact the elderly psychologically and produce or deepen their sense of loneliness. Based on the above conclusions, this paper further puts forward relevant policy suggestions from three aspects: constructing a disease prevention and control system for the rural elderly, improving the care and service system for the rural elderly, reshaping rural filial piety culture, and creating a good atmosphere of “respecting, loving and respecting parents” in rural areas.

## 1. Introduction

With the outflow of the rural labor force and the growing elderly population, the traditional pension model has been seriously impacted, and the lack of support for the elderly has become an increasingly important social phenomenon. Under this background, the living conditions and health status of the rural elderly have attracted more and more social attention. In 2019, No. 1 central document, the CPC Central Committee, and the State Council, issued several opinions on adhering to the priority of agriculture and rural development and doing well in the work of “three rural issues”. The document emphasized the governance of the bad social atmosphere such as the decline of filial piety and the lack of support for the elderly, and the establishment of a caring service system for the elderly in rural areas [1]. Under the background of the continuous evolution of urbanization, it is particularly urgent to care for the rural elderly. According to the development law of individual life cycle, with aging, the elderly will experience weakening physical function, the probability of disease will increase accordingly, and their health status will decline. In addition, the labor migration of children and grandchildren and other events have formed the special physiological and psychological characteristics of the rural elderly. Previous news reports and academic research mostly focused on the physical health of the rural elderly. However, in recent years, depression and suicide of the rural elderly have become more common, and the public have begun to pay more attention to this problem. Based on this, the academic community began to conduct in-depth research on the mental health of the rural elderly.

The dual pressure of rural labor outflow and population aging has led to the increasing prominence and attention of the rural elderly population. Academic research on the rural elderly is also increasing. At present, research on the rural elderly mainly focuses on the following directions: first, the subjective welfare and health of the rural elderly [2,3,4,5]; second, the willingness and mode of providing for the elderly in rural areas [6,7]; third, the intergenerational relationship between the rural elderly and their children [8,9,10,11,12,13,14]. Among them, research on the mental health problems of the rural elderly is mostly discussed from the level of children and does not deeply explore whether the elderly’s diseases and other self-factors will produce loneliness and bring psychological problems [15,16,17,18,19]. From the perspective of empirical research, it is suggested that the illness of the elderly will produce or aggravate the sense of loneliness. On the one hand, it is the pain, helplessness and loneliness directly caused by the disease. On the other hand, the disease leads to an increase in family burden, resulting in a change in family strategy and intergenerational relationship. In rural areas with serious aging, clarifying whether old-age diseases will bring loneliness and its underlying mechanism can prevent the disease from producing lonely emotions and the vicious circle of new diseases caused by loneliness, to alleviate the health problems of rural elderly people.

By combing the existing literature, it is not difficult to find that there are few studies on the loneliness of the elderly in rural areas. At present, a few studies in this area mostly analyze the loneliness of the elderly and explore its influencing factors [20,21,22]. Among them, influencing factors such as age, education level and self-rated health of the elderly will influence their loneliness [23]. At the same time, some influencing factors from the family will also affect the generation of loneliness, such as family living style, children’s intergenerational support, etc. [24,25]. In addition, the influencing factors at the social level such as social support and socio-economic deprivation will also affect their loneliness [26,27,28]. Most of these studies ignore the influencing factors such as diseases from the elderly themselves, which means that current research on the loneliness of the elderly in rural areas lacks persuasion. With the aging of elderly individuals, their physical function begins to weaken, their health status begins to decline, and the risk of “geriatric diseases” and other diseases increases. The decline in their physical health level may lead to loneliness and other negative psychological feelings [29,30,31]. At the same time, the outflow of the rural young and middle-aged labor force has led to changes in the traditional rural family living mode and intergenerational coexistence mode. Mobility and left behind have a dual impact of increasing space and reducing time [32,33,34]. Under this realistic background, when the elderly have physical diseases, their care problems and medical burden break the dynamic balance and intergenerational coexistence mode between mobility and left behind, increasing the risk of the elderly being ignored or even abandoned, and may further deepen the loneliness of the elderly and fall into a vicious circle. To prevent the occurrence of such problems, this paper will explore the relationship between disease and loneliness of the rural elderly, whether disease has an impact on the loneliness of the rural elderly and further explore the underlying mechanisms to put forward relevant suggestions to prevent the elderly from falling into the dual dilemma of disease and loneliness.

We undertake a theoretical analysis from three aspects: the concept of “filial piety”, the theory of population migration and the “serious disease effect”. First of all, from the perspective of the concept of “filial piety”, in China, the “back feeding pension” has a long history and has many cultural traditions that maintains its continuation and development. As an important part of Confucian culture, the concept of “filial piety” has a profound impact on Chinese society. “The widower, the widow, the orphan and the childless are supported”, “When parents are ill, children taste medicine first and serve day and night without leaving his bed”. This traditional culture of supporting parents requires parents to take care of their sick children. When the elderly suffer from diseases, “filial piety culture” and “back feeding pension tradition” may make children pay more attention to the health of the elderly, improving the intergenerational relationship between the rural elderly and their children with cultural and traditional education and constraints, so as to reduce the risk of loneliness and other negative psychological feelings of the elderly due to the decline in their physical health level [35,36]. In short, the disease may alleviate the loneliness of the rural elderly.

Secondly, according to the theory of population migration, the flow of the rural young and middle-aged labor force to cities is based on the rational consideration of maximizing personal interests [37]. The flow of the rural young and middle-aged labor force to the city emphasizes the role of social labor and weakens the family role in the original family, reducing the time and energy to care for and communicate with parents, which means that the elderly’s emotional and behavioral dependence on their children is hindered by time and space under the opposition between mobility and left behind [38,39]. When the emotional sustenance and care dependence of the rural elderly on their children are limited, they may feel a sense of loss and loneliness. In this reality, when the elderly suffer health problems again, it may aggravate their own loneliness. At the same time, the elderly suffering from diseases may increase the burden on their family, change family strategies and intergenerational relations, and deepen the elderly’s loneliness [40].

The third is based on the theoretical analysis of “serious disease effect”. The World Health Organization defines health as physical health, mental health and social adaptation. Physical health and mental health are indicators of health. Their relationship may be complementary. The quality of physical condition will affect mental health. On the other hand, because of diseases, physical diseases will distort people’s psychology, resulting in inferiority complex, resentment, depression and other changes [17]. That is, the quality of physical condition will affect mental health. The elderly are more likely to become sick due to their age and physical aging, which may further affect their mental health and produce loneliness, depression and other emotions. However, for the elderly, especially the rural elderly, whether their physical health will affect their mental health, and if so through what mechanism, these problems need to be addressed. Some scholars believe that physical health will affect mental health through diseases, mainly through the types of diseases, especially chronic diseases. However, in addition to the types of diseases, the severity of diseases may also have an impact on the rural elderly [22]. When the chronic diseases of the rural elderly develop into serious diseases or direct sudden serious diseases, their action ability is impaired or their fear of death will affect their psychological subjective consciousness, resulting in loneliness and other negative psychological feelings, which can be understood as “serious disease effect”. Based on the above, we can make the following research hypothesis:

**Hypothesis** **1** **(H1).** *The physical health status of the elderly in rural areas will significantly affect their loneliness.*

**Hypothesis** **2** **(H2).** *The influence of the health status of the rural elderly on their loneliness is realized through the “serious illness effect”.*

## 2. Data and Methods

### 2.1. Data Sources

The data used in this paper are the health status survey data of China’s elderly population (CLHLS), which are micro data. The data were obtained through a large-scale survey jointly conducted by the China Economic Research Center of Peking University, the aging health and family research center of Peking University and Duke University in China. The data adopt probability proportional to size sampling. Since the baseline survey in 1998, there have been seven large-scale surveys. This paper selected the latest survey data of 2014 published in 2018, which covers more than 7000 subjects from 23 provinces (autonomous regions and municipalities directly under the central government). The survey mainly includes the respondents’ family situation, economic status, living conditions, health status and social welfare level. CLHLS database has a large sample size. According to the research needs of this paper, we directly eliminated invalid samples and samples with missing key variables.

### 2.2. Methods

#### 2.2.1. Selection and Definition of Model Variables

The explanatory variable of this paper is the loneliness of the rural elderly. The concept of loneliness usually represents the subjective feeling of being excluded by others or a group in the field of social psychology. Loneliness is an individual’s feeling about the quantity and quality of their own social communication, which may be the psychological feeling produced by individuals being alone for a long time [41,42]. Loneliness, as one of the most common psychological problems of the rural elderly, has always been the focus of academic attention [21]. With the aggravation of China’s aging, the health problems of the elderly population are paid more and more attention. The health problems of the elderly are not only reflected in the physiological aspects, but also in the psychological aspects. As an important indicator of mental health, loneliness is not only a reflection of the mental health status of the rural elderly, but also a sensitive indicator of depression and suicidal ideation [17]. This paper selects the loneliness of the rural elderly as the explanatory variable. The variable is defined as “do you often feel lonely?” the elderly often feel lonely, and the value is 1, otherwise the value is 0.

There are many kinds of diseases with complex definitions and there are many overlaps between major diseases and chronic diseases. In order to select the proxy variables of diseases, more diseases of the elderly are included and the availability of data is considered. Based on relevant research, this paper selects the comprehensive index of self-rated health variable, which is often used in academia and is relatively easy to obtain, as the explanatory variable in the benchmark regression. If the self-rated health status is bad or very bad, the value is 0, if on the contrary, the value is 1.

In order to more accurately analyze the impact of health status on the loneliness of the rural elderly, this paper controls the individual and family characteristics of the elderly. The control variables mainly include the individual characteristic variables such as age, gender, education level and personality of the elderly and the family characteristic variables such as family income, intergenerational support and the number of children. The age and gender of the elderly are the specific age and gender of the sample elderly, and the education level is the specific years of education of the elderly. The character of the elderly is defined as “can you think about everything?”, that is, they are cheerful and assigned a value of 1, otherwise they are assigned a value of 0. Among the family characteristic variables, family income is the logarithm of the actual income of the family. Children’s visit is defined as whether the children of the elderly often visit themselves. The value of frequent visit is 1 and the value of infrequent visit is 0. The number of children is the number of children raised by the elderly. The specific variable definitions and values are shown in Table 1.

#### 2.2.2. The Models

The purpose of this study is to explore the relationship between disease and loneliness of the rural elderly. The dependent variable is the loneliness of the rural elderly, which is a qualitative binary variable; that is, often feel lonely or not often feel lonely. A logistic model is suitable for the analysis of binary variables, so this study selected a logistic model for regression analysis.

The form of logistic probability function is:

(1) $$P=\frac{Exp(Z)}{1+Exp(Z)}$$

*Z* is the linear combination of variables *X1, X2, X3... X*:

(2) $$Z=b_{0}+b_{1}X_{1}+b_{2}X_{2}+\cdot \cdot \cdot +b_{n}X_{n}=b_{0}+\sum_{i=1}^{n}b_{i}X_{i}$$

In the process of data statistical analysis, if the rural elderly often feel lonely it is set as *P* (y = 1); the probability of less and never feeling lonely is 1 − *P* (y = 0). In logistic regression analysis, *P* is usually transformed into:

(3) $$LogitP=\ln(\frac{P}{1-P})=b_{0}+\sum_{i=1}^{n}b_{i}X_{i}$$

In this way, the linear expression between the function of probability and the independent variable is obtained. The independent variable y is the loneliness of the rural elderly. If the elderly often feel lonely, the dependent variable is assigned as 1; if they do not feel lonely often (less or never), the dependent variable is assigned 0. The explanatory variables of individual characteristics and family characteristics form a linear combination Z. In this paper, Stata13.0 software is used as the statistical tool.

## 3. Results

### 3.1. Descriptive Statistics of the Variables

According to the research needs of this paper, we directly eliminated invalid samples and samples with missing key variables, and finally obtained 4789 samples. Table 2 makes descriptive statistics of relevant variables according to the definition and assignment of variables in Table 1. It can be seen from Table 2 that more than 1/3 of the sample elderly often feel lonely, 14.39% of the rural elderly have impaired health status, the age of the sample elderly is generally too old, and the oldest sample elderly is up to 117 years old. Calculated by age, most of the sample elderly were born before the founding of the people’s Republic of China. Because of the age background, the education level is generally low. At the same time, more than 2/3 of the rural elderly in the sample are cheerful and can think about everything. The average family income of the elderly in the sample is about 16,521.21, with a maximum of 100,000 yuan and a minimum of 300 yuan. In terms of the number of children and intergenerational support, the average number of children owned by the sample elderly is about 4.6087, including at least one and at most 15. 97.2% of the rural elderly often have children to visit to provide intergenerational support.

### 3.2. Benchmark Regression Results

In this paper, stata13.0 software is used to carry out the benchmark regression of the impact of self-rated health status of rural elderly on loneliness, and the control variables from individual characteristics and family characteristics are added to observe whether the regression results are robust. Overall, the model runs well, and the influence direction and significance level of variables do not change significantly among the regression results. Table 3 shows the estimation results of the model. In Table 3, Model 1 only investigates the impact of the physical health status of the elderly on loneliness, that is, the impact of disease on loneliness. Model 2 adds control variables from the individual characteristics of the elderly such as age, gender, education level and personality based on model 1, and Model 3 adds the logarithm of family income, intergenerational support, the number of children and other control variables to measure family characteristics. In the three models, the core variable, physical health status of the elderly, has a significant positive impact on loneliness. The elderly with impaired physical health are more likely to feel lonely; that is, disease will bring loneliness or deepen loneliness. The possible explanation is that due to the gap between urban and rural development and the massive outflow of the rural young and middle-aged labor force, the traditional living mode and the care mode of the rural elderly have been broken, resulting in the opposition between rural children’s mobility and their parents’ remain. Compared with the traditional way of living with their children’s parents, communication between the rural elderly and their children has been reduced and the constraints of clan relatives and neighbors on their children’s “filial piety” are also weakened. The physical function of the elderly decreases and the risk of disease increases. When the physical health condition is damaged, it will bring their own sadness and loss. In addition, there may be the risk of neglect and abandonment of their children, so that the disease will bring loneliness or deepen the sense of loneliness to the rural elderly.

At the same time, model two and model three show that the age, gender, personality, family income and age support of children in the individual characteristics and family characteristics have significant effects on the loneliness of the elderly. Among them, the age of older persons has a significant positive effect on their loneliness. The older the elderly, the older the elderly are, the more likely they are to feel lonely. The possible reason is that the older the elderly, the higher the risk of illness, and the negative emotions brought by the disease also increase. At the same time, with the growth of age, the elderly’s fear of death deepens, coupled with the absence of children around them, which deepens the loneliness of the elderly.

Gender has a significant negative impact on the loneliness of the elderly: rural elderly women are more likely to feel lonely than men. The possible explanation is that in China’s rural areas, there is a family division tradition of “men outside and women inside”. Rural women mostly take the family as the focus of their life and spiritual sustenance. When they age, their sons “separate” or go out and the marriage of the daughter changes the focus and maintenance of the family, which is a female emotion and is more likely to fall into loss and loneliness. In comparison, the male elderly may have richer social relations and entertainment methods to disperse the spiritual sustenance and alleviate the sense of loneliness, so their sense of loneliness is weaker than that of women. The cheerful old people can think about everything. Compared with the non-cheerful old people, they can alleviate their loneliness through self-enlightenment. The logarithm of family income has a significant negative impact on the loneliness of the elderly; that is, the higher the family income, the lower the possibility of the elderly feeling lonely. The possible reason is that the higher the family income, the elderly may have more to spend on entertainment services to alleviate their loneliness. Intergenerational support also has a significant negative impact on the loneliness of the elderly. The elderly with intergenerational support; that is, the elderly with children who often visit, are less likely to feel lonely.

### 3.3. ”Serious Illness Effect” of Loneliness of Rural Elderly

Based on the binary logistic model, this paper discusses the impact of the physical health status of the rural elderly on their loneliness and finds that health status has a significant impact on the occurrence of loneliness of the rural elderly. However, the above research only investigated the impact of the elderly’s self-rated physical health on their loneliness and did not discuss the severity and types of diseases, which makes the research relatively thin and difficult to describe the impact mechanism of diseases on loneliness. Therefore, this paper further discusses, based on the above research contents, the core variable of physical health status of the elderly, which is replaced by the type and severity of the disease. Based on the incidence rate, the main diseases in this study are five types of common chronic diseases, including hypertension, diabetes, heart disease, cerebrovascular disease, and respiratory diseases. The severity of disease is the severity of disease that requires hospitalization or if they are completely bedridden at home. The definition and descriptive statistics of specific disease types are shown in Table 4.

Table 4 is a sample of elderly people suffering from hypertension, diabetes, heart disease, cerebrovascular diseases, respiratory diseases, and other major diseases. From Table 4, we can see that the most common diseases of the elderly are hypertension, heart disease and respiratory diseases (mainly bronchitis, emphysema, asthma, or pneumonia). Among them, the incidence rate of hypertension is the highest at 30.72%, and the incidence rate of heart disease and respiratory disease is about 10%. At the same time, the sample elderly had a high probability of serious illness requiring hospitalization or forcing them to be bedridden at home, as high as 25.85%.

Table 5 presents the estimation results of hypertension, diabetes, heart disease, cerebrovascular diseases, respiratory diseases, and major diseases as the core variables in Formula (1). From Table 5, we can see those chronic diseases such as hypertension, diabetes, heart disease, cerebrovascular diseases and respiratory diseases have no significant effect on loneliness of the elderly in rural areas. However, major diseases have a significant impact on the loneliness of the elderly. Combined with the previous theoretical analysis and model estimation results, the possible explanation is that the illness of the elderly in rural areas has a significant impact on their loneliness, but generally, chronic diseases have little impact on the loneliness of the elderly. However, when the development of chronic diseases causes serious diseases requiring hospitalization or bedridden at home, in these instances, the disease will have a significant positive impact on the loneliness of the elderly, which can be understood as the “serious disease effect”; that is, when the chronic disease of the elderly in rural areas develops into a serious disease or a direct sudden serious disease, their action ability is impaired, or their fear of death will have an impact on their psychological subjective consciousness, loneliness and other negative psychological feelings, resulting in loneliness, At the same time, intergenerational support also has a significant impact on the loneliness of the elderly. When the elderly is suffering from a serious illness requiring hospitalization or bedridden at home, the dual pressure of economic burden and care burden may make children regard their parents as a “burden”, reducing visiting behavior and deepening the loneliness of the elderly.

### 3.4. Robustness Test

To solve the endogenous problem caused by sample selection deviation and other reasons, this paper further tests the robustness of the above regression findings on the impact of the health status of the elderly on loneliness and the impact of major diseases on loneliness. Therefore, this paper uses the propensity value matching method (PSM) to take the elderly with “undamaged health status” as the treatment group in other areas, selects appropriate samples from the samples with “impaired health status” for matching to solve the possible impact of the above problems [34]. Similarly, this paper takes the elderly without “major diseases” as the treatment group, and selects appropriate samples from other samples with “major diseases” for matching.

The specific operations are as follows: firstly, according to the observable individual and family characteristics (this paper mainly selects the control variables in the previous article: age, gender, education level, personality, logarithm of family income, intergenerational support and number of children), the probability of impairment of health status of the elderly is estimated, and the tendency score is calculated, which can be expressed as:

(4) $$P(X_{i})=\mathrm{Pr}(F_{i}=1|X_{i})=\frac{\exp(\beta X_{i})}{1+\exp(\beta X_{i})}+\varepsilon$$

The binary dummy variable in the formula is expressed as the health status of the rural elderly is impaired (or suffering from major diseases), the influencing factors of the health status of the rural elderly are impaired (or suffering from major diseases), the coefficient of the model and the random error. Then, the elderly without impaired health status (or without major disease) who have the closest tendency score to the rural elderly with impaired health status (or with major disease) are taken as the counterfactual. Then, the difference in the probability of loneliness between the two groups is compared, and the average value of the calculated difference is taken into account to obtain the average effect of health status on the loneliness of rural elderly. This can be expressed as:

(5) $$ATT=E(Y_{i,1}|T_{i}=1)-E(Y_{i,0}|T_{i}=1)$$

In this paper, neighbor matching, caliper matching, kernel matching, and other matching methods are selected. The samples without impaired health status (or without major diseases) are taken as the control group, and the samples with impaired health status (or with major diseases) are taken as the treatment group. Through matching, the comparability between the treatment group and the control group is ensured. Among the 4789 observation samples in total, 18 in the treated group are not in the common value range (off support), and the other observation values are in the common value range (on support). The changes in characteristic variables before and after sample matching obtained by three matching methods are shown in Table 6, Table 7 and Table 8. Based on the existing research, the absolute value of the standard deviation after matching is usually equal to 10 as the judgment standard of the matching effect. If the absolute value of the standard deviation after matching is less than 10, the matching effect is better [34]. It can be seen from Table 6, Table 7 and Table 8 that before matching, the average standard deviation of characteristic variables is greater than 10, and there are obvious differences between groups, which will cause estimation deviation. After matching, the standard deviation of sample characteristic variables of the two groups is significantly reduced and the absolute value of standard deviation is less than 10, indicating that the mean difference of each characteristic variable is very small, the feature difference between samples is eliminated to a certain extent, and the matching effect is good. Taking the samples without major diseases as the control group and the samples with major diseases as the treatment group, the changes in characteristic variables before and after sample matching are similar after using neighbor matching, caliper matching (radius), quadratic kernel matching and other matching methods, which are not described one by one due to the length of the article.

To ensure the robustness of the estimation results, this paper uses neighbor matching, caliper matching and quadratic kernel matching to investigate the average processing effect of the health status of the rural elderly on their loneliness. The estimated results of average treatment effect in Table 9 show that before matching, the health status of the elderly at the significance level of 1% increases the incidence of loneliness of the elderly in rural areas by about 50%. After matching, the effect of health status of rural elderly on the deepening of loneliness is still significant, and the deepening degree converges to the range of 35% to 45%, which shows that health status has a significant impact on the occurrence of loneliness of the rural elderly. Therefore, hypothesis 1 (H1) is verified.

To ensure the robustness of the estimation results, this paper uses neighbor matching, caliper matching and quadratic kernel matching to investigate the average treatment effect of major diseases on loneliness rural elderly people. The average treatment effect estimation results in Table 10 show that before matching, the prevalence of serious diseases of the elderly increased the incidence of loneliness of the elderly in rural areas by 41.92% at the significance level of 1%. After matching, the effect of the health status of the rural elderly on the deepening of their loneliness is still significant, and the deepening degree converges to about 37%, which shows that the health status has a significant impact on the occurrence of loneliness in the rural elderly, and the relevant conclusions on the “serious disease effect” obtained above are persuasive. Therefore, hypothesis 2 (H2) is verified.

## 4. Discussion

According to the comprehensive benchmark regression results and robustness test results, it can be found that the health status of rural elderly has a significant positive impact on their loneliness; that is, the elderly with physical health problems are more likely to feel lonely than the elderly without physical health problems. With further exploration, we can see that there is no significant difference in the loneliness of rural elderly people with different diseases, such as hypertension, diabetes, heart disease, cerebrovascular diseases, and respiratory diseases, but significant diseases have a significant impact on loneliness of the elderly. It can be considered that illness in the rural elderly has a significant impact on their loneliness [23], which often has a “serious disease effect”. This allows both H1 and H2 to be verified.

What is the “serious illness effect”? As mentioned in the previous theoretical analysis, simple chronic diseases have little impact on the loneliness of the rural elderly, but when the development of chronic diseases causes serious diseases requiring hospitalization or the elderly to be bedridden at home, the disease will have a significant impact on the loneliness of the elderly. The reason for this “serious disease effect” may be that when the chronic disease of the rural elderly develops into a serious disease or a direct sudden serious disease, their action ability is impaired, or their fear of death will affect their psychological subjective consciousness, resulting in loneliness and other negative psychological feelings, and then produce loneliness. At the same time, intergenerational support also has a significant impact on the loneliness of the elderly. When the elderly is suffering from a serious illness requiring hospitalization or them to be bedridden at home, the dual pressure of economic burden and care burden may make children regard their parents as a “burden”, reducing visiting behavior and deepening the loneliness of the elderly. Currently, in China, the loneliness of the elderly and its “serious disease effect” may not only appear in rural areas, but may also emerge due to the increase in empty nesters in cities, with their loneliness and other mental health problems becoming more and more obvious [38]. Of course, the characteristics and influencing factors of urban related problems also need to be further studied by scholars.

## 5. Conclusions and Recommendations

Based on the data from China’s elderly population health survey (CLHLS) in 2014, this paper discusses the impact of the health status of the elderly on loneliness in rural areas and explores the mechanisms of influence. It also explores the effects of different kinds of diseases, such as hypertension, diabetes, heart disease, cerebrovascular diseases, respiratory diseases, and so on, on the loneliness of the elderly in rural areas. (PSM) tested the robustness of correlation regression findings and obtained the following main conclusions. First, the health status of the rural elderly has a significant positive impact on their loneliness; that is, the rural elderly with poor health are more likely to experience loneliness. At the same time, the age of the elderly has a significant positive impact on their loneliness. On the contrary, gender, personality, family income and intergenerational support of the elderly have a negative impact on their loneliness. Second, chronic diseases such as hypertension, diabetes, heart disease, cerebrovascular diseases, respiratory diseases and other chronic diseases have no significant impact on the loneliness of the elderly in rural areas. However, when the rural elderly’s chronic diseases develop into serious diseases or acute serious diseases, it will affect their subjective psychological feelings, and they will experience loneliness and other negative psychological feelings, which can produce or deepen loneliness. This is the “serious illness effect” of loneliness of the elderly in rural areas.

The above conclusions are of great significance for the prevention and control of physical and psychological diseases in the rural elderly. Combined with the full text, we can develop corresponding policies to address the above issues, focusing on the following three aspects. First, we can build a disease prevention and control system for the rural elderly, which will prevent them from suffering from chronic diseases without knowing themselves or help them to understand the severity of their disease through free physical examination and chronic disease screening. This would also help to promote the “rural family doctor” and “general practitioner special post plan” on the basis of improving the existing “new rural cooperative medical system” and “serious disease insurance”, in addition to other relevant medical policies Additionally, other convenient medical services will ensure that diseases in the elderly can be diagnosed and treated timely and effectively to prevent them from developing into serious diseases requiring hospitalization or them to be bedridden at home. This will help reduce loneliness and other negative effects on their subjective psychological emotions, resulting in the dual repression of physical and psychological diseases [43]. Second, we can improve the care service system for the rural elderly: the village organization or community establishes and effectively uses the elderly activity room to organize relevant cultural and recreational activities and focuses on the life and health status of the rural elderly, whose children are away all year round and whose health is damaged. While the village organization or community provides relevant services, it creates “mutual assistance between villagers and care for the elderly” to alleviate loneliness in the rural elderly with cultural and recreational activities and spiritual comfort among rural neighbors. This also reduces the risk of depression and other mental diseases and suicide [44,45]. Third, we can reshape the rural filial piety culture and emphasize the idea that one should “respect, love and honor parents”. At present, family pension is still the main pension in rural areas and the attitude of children plays a vital role in the quality of life and health level of the rural elderly. Relevant departments should deal with the current trend of declining filial piety and lack of support for the elderly in some rural areas and launch a number of rural filial piety spiritual civilization construction demonstration counties throughout the country. In the demonstration counties, select the civilized villages and towns with filial piety, the most beautiful families and model individuals with filial piety to their parents, excavate and set up moral models, give play to the role of demonstration and guidance, and create a good atmosphere. And promote the stability of children’s support to their parents in the form of rural social network constraints and filial piety cultural norms. This will ensure that the rural elderly can really have a sense of security, dependence and joy as they age.

## Figures and Tables

**Table 1:** Variable definition and assignment.

Variable Type		Variable Name	Variable Definition
explained variable		loneliness status	often feel lonely = 1; Less or never feel lonely = 0
core explanatory variable		physical health	physical health status troubled by disease = 1; No disease = 0
control variable	individual characteristics	age	actual age of the elderly in the surveyed year
		gender	male = 1; 0 = female = 0
		education level	actual years of education of the elderly
		character	cheerful personality = 1; Not cheerful = 0
	family characteristics	logarithm of household income	logarithm of the actual income of the elderly’s family
		intergenerational support	frequent visits with children = 1; Infrequent visits by children = 0
		number of children	number of children raised by the elderly

**Table 2:** Statistical results of variables.

Variable Type		Variable Name	Mean Value	Standard Deviation	Minimum Value	Maximum Value
Explained Variable		Loneliness status	0.3828	0.4861	0	1
Core explanatory variable		Physical health	0.1439	0.3510	0	1
Control Variable	Individual characteristics	Age	85.2063	10.4690	60	117
		Gender	0.4548	0.2480	0	1
		Education level	2.0710	3.1619	0	20
		Character	0.7135	0.4522	0	1
	Family characteristics	Logarithm of household income	9.7124	1.2875	5.7038	11.5129
		Intergenerational support	0.9720	1.2875	0	1
		Number of children	4.6087	0.1649	1	15

**Table 3:** Model estimation results of health status on loneliness of rural elderly.

Variable Type	Variable Name	Model 1		Model 2		Model 3	
		Coef	OR	Coef	OR	Coef	OR
Core variable	Physical health	0.675 ***	1.9649	0.397 ***	1.4869	0.379 ***	1.4606
		(0.0829)		(0.0951)		(0.0958)	
Personal characteristics	Age			0.0342 ***	1.0347	0.0353 ***	1.0359
				(0.00334)		(0.00342)	
	Gender			−0.210 ***	0.8108	−0.233 ***	0.7923
				(0.0737)		(0.0741)	
	Education level			−0.0190	0.9811	−0.0126	0.9875
				(0.0129)		(0.0130)	
	Character			−1.527 ***	0.2171	−1.523 ***	0.2181
				(0.0702)		(0.0706)	
Family characteristics	Logarithm of household income					−0.101 ***	0.9041
						(0.0256)	
	Intergenerational support					−0.360 *	0.6978
						(0.198)	
	Number of children					−0.0143	0.9858
						(0.0176)	
	Constant term	−0.580 ***	0.5601	−2.280 ***	0.1023	−0.983 **	0.3742
		(0.0326)		(0.306)		(0.434)	
	Observed value	4789		4789		4789	
	R-squared	0.0104		0.1247		0.1279	

Note: Coef represents the coefficient, the standard error is in parentheses, and OR represents the odds ratio. ***, ** and *, respectively indicate that the explanatory variable coefficients are significant at the levels of 1%, 5% and 10%.

**Table 4:** Definition and description of disease type variables and statistical results.

Disease Type	Variable Definition	Mean Value	Standard Deviation	Minimum Value	Maximum Value
Hypertension	Now suffering from hypertension = 1 No hypertension now = 0	0.3072	0.4614	0	1
Diabetes	Suffering from diabetes mellitus = 1 No diabetes = 0	0.0432	0.2034	0	1
Heart disease	Now suffering from heart disease = 1 No heart disease now = 0	0.1109	0.3140	0	1
Cerebrovascular diseases	Now suffering from cerebrovascular disease = 1; Conversely = 0	0.0785	0.2690	0	1
Respiratory disease	Now suffering from respiratory diseases = 1; Conversely = 0	0.1069	0.3090	0	1
Major diseases	Serious illness requiring hospitalization or bedridden at home in recent two years = 1 Serious illness in recent two years = 0	0.2585	0.4379	0	1

**Table 5:** Model estimation results of health status on loneliness of rural elderly.

Variable Name	Model 4		Model 5		Model 6		Model 7		Model 8		Model 9	
Hypertension	0.00256	1.0026										
	(0.0719)											
Diabetes			−0.207	0.8134								
			(0.169)									
Heart disease					0.0805	1.0839						
					(0.103)							
Cerebrovascular diseases							0.132	1.1412				
							(0.124)					
Respiratory disease									−0.0839	0.9195		
									(0.108)			
Major diseases											0.157 **	1.1694
											(0.0732)	
Age	0.0344 ***	1.0350	0.0341 ***	1.0347	0.0346 ***	1.0352	0.0347 ***	1.0352	0.0343 ***	1.0349	0.0348 ***	1.0354
	(0.00343)		(0.00342)		(0.00341)		(0.00341)		(0.00341)		(0.00341)	
Gender	−0.237 ***	0.7892	−0.240 ***	0.7866	−0.235 ***	0.7908	−0.239 ***	0.7875	−0.234 ***	0.7914	−0.241 ***	0.7856
	(0.0739)		(0.0739)		(0.0739)		(0.0739)		(0.0739)		(0.0740)	
Education level	−0.0132	0.9869	−0.0129	0.9872	−0.0132	0.9869	−0.0132	0.9869	−0.0131	0.9870	−0.0129	0.9872
	(0.0130)		(0.0130)		(0.0130)		(0.0130)		(0.0130)		(0.0130)	
Character	−1.571 ***	0.2077	−1.573 ***	0.2075	−1.571 ***	0.2078	−1.566 ***	0.2088	−1.572 ***	0.2076	−1.562 ***	0.2097
	(0.0697)		(0.0697)		(0.0697)		(0.0699)		(0.0697)		(0.0698)	
Logarithm of household income	−0.105 ***	0.9006	−0.104 ***	0.9013	−0.105 ***	0.9003	−0.105 ***	0.9004	−0.104 ***	0.9008	−0.106 ***	0.8994
	(0.0255)		(0.0255)		(0.0255)		(0.0255)		(0.0255)		(0.0255)	
Intergenerational support	−0.385 *	0.6802	−0.382 *	0.6825	−0.387 **	0.6791	−0.381 *	0.6830	−0.384 *	0.6810	−0.376 *	0.6865
	(0.197)		(0.197)		(0.197)		(0.197)		(0.196)		(0.196)	
Number of children	−0.0123	0.9877	−0.0125	0.9876	−0.0123	0.9877	−0.0127	0.9874	−0.0117	0.9884	−0.0148	0.9854
	(0.0176)		(0.0176)		(0.0176)		(0.0176)		(0.0176)		(0.0177)	
Constant term	−0.766 *	0.4649	−0.736 *	0.4788	−0.786 *	0.4556	−0.797 *	0.4509	−0.755 *	0.4699	−0.826 *	0.4376
	(0.430)		(0.429)		(0.429)		(0.429)		(0.429)		(0.429)	
Observed value	4789		4789		4789		4789		4789		4789	
R-squared	0.1253		0.1255		0.1254		0.1254		0.1254		0.1260	

Note: Coef represents the coefficient, the standard error is in parentheses, and OR represents the odds ratio. ***, ** and *, respectively indicate that the explanatory variable coefficients are significant at the levels of 1%, 5% and 10%.

**Table 6:** Changes in characteristic variables before and after sample matching (neighbor).

		Mean Value		Standard Deviation (%)	Deviation Reduction (%)	*t*-Test	
Variable	Sample	Interactive Group	Control Group			*t*-Value	*p*-Value
Age	Before matching	84.7300	85.286	−5.4	40.9	−1.29	0.197
	After matching	84.7300	85.059	−3.2		−0.60	0.550
Gender	Before matching	0.4122	0.4620	−10.0	88.3	−2.43	0.015
	After matching	0.4122	0.4180	−1.2		−0.22	0.827
Education level	Before matching	1.8200	2.1132	−9.6	97.8	−2.25	0.024
	After matching	1.8200	1.8135	0.2		0.04	0.967
Character	Before matching	0.5080	0.7481	−51.3	98.8	−13.12	0.000
	After matching	0.5080	0.5110	−0.6		−0.11	0.914
Logarithm of household income	Before matching	9.5360	9.7420	−15.7	90.5	−3.89	0.000
	After matching	9.5360	9.5165	1.5		0.27	0.786
Intergenerational support	Before matching	0.9550	0.9749	−10.8	52.5	−2.93	0.003
	After matching	0.9550	0.9456	5.1		0.81	0.421
Number of children	Before matching	4.7271	4.5888	7.5	83.5	1.82	0.069
	After matching	4.7271	4.7500	−1.2		−0.22	0.823

**Table 7:** Changes of characteristic variables before and after sample matching (Radius).

		Mean Value		Standard Deviation (%)	Deviation Reduction (%)	*t*-Test	
Variable	Sample	Interactive Group	Control Group			*t*-Value	*p*-Value
Age	Before matching	84.730	85.286	−5.4	49.6	−1.29	0.197
	After matching	84.737	85.017	−2.7		−0.51	0.609
Gender	Before matching	0.4122	0.4620	−10.0	97.9	−2.43	0.015
	After matching	0.4134	0.4144	−0.2		−0.04	0.969
Education level	Before matching	1.8200	2.1132	−9.6	93.1	−2.25	0.024
	After matching	1.8253	1.8050	0.7		0.13	0.897
Character	Before matching	0.5080	0.7481	−51.3	100.0	−13.12	0.000
	After matching	0.5095	0.5094	0.0		0.00	0.999
Logarithm of household income	Before matching	9.5360	9.7420	−15.7	83.5	−3.89	0.000
	After matching	9.5415	9.5754	−2.6		−0.47	0.639
Intergenerational support	Before matching	0.9550	0.9749	−10.8	68.5	−2.93	0.003
	After matching	0.9578	0.9515	3.4		0.56	0.578
Number of children	Before matching	4.7271	4.5888	7.5	60.3	1.82	0.069
	After matching	4.7205	4.7754	−3.0		−0.53	0.594

**Table 8:** Changes in characteristic variables before and after sample matching (Kernel).

		Mean Value		Standard Deviation (%)	Deviation Reduction (%)	*t*-Test	
Variable	Sample	Interactive Group	Control Group			*t*-Value	*p*-Value
Age	Before matching	84.7300	85.286	−5.4	30.9	−1.29	0.197
	After matching	84.7300	85.115	−3.8		−0.70	0.482
Gender	Before matching	0.4122	0.4620	−10.0	74.7	−2.43	0.015
	After matching	0.4122	0.4248	−2.5		−0.47	0.636
Education level	Before matching	1.8200	2.1132	−9.6	89.7	−2.25	0.024
	After matching	1.8200	1.8502	−1.0		−0.19	0.848
Character	Before matching	0.5080	0.7481	−51.3	97.5	−13.12	0.000
	After matching	0.5080	0.5140	−1.3		−0.22	0.824
Logarithm of household income	Before matching	9.5360	9.7420	−15.7	60.9	−3.89	0.000
	After matching	9.5360	9.6166	−6.1		−1.13	0.260
Intergenerational support	Before matching	0.9550	0.9749	−10.8	68.9	−2.93	0.003
	After matching	0.9550	0.9612	−6.1		−0.57	0.568
Number of children	Before matching	4.7271	4.5888	7.5	95.7	1.82	0.069
	After matching	4.7271	4.7212	0.3		0.06	0.953

**Table 9:** Estimation results of average treatment effect of health status of rural elderly on their loneliness.

Matching Method	Sample	Treated	Controls	ATT Difference (Difference)	S.E.	*t*-Value
Neighbor	Before matching	0.5239	0.3590	0.1649	0.0199	8.30 ***
	After matching	0.5239	0.4510	0.0729	0.0234	3.12 ***
Radius	Before matching	0.5239	0.3590	0.1649	0.0199	8.30 ***
	After matching	0.5239	0.4474	0.0751	0.0212	3.55 ***
Kernel	Before matching	0.5239	0.3590	0.1649	0.0199	8.30 ***
	After matching	0.5239	0.4440	0.0800	0.0210	3.81 ***

Note: ***, respectively indicate that the explanatory variable coefficients are significant at the levels of 1%.

**Table 10:** Estimated results of average treatment effect of major diseases on loneliness of rural elderly.

Matching Method	Sample	Treated	Controls	ATT Difference (Difference)	S.E.	*t*-Value
Neighbor	Before matching	0.4192	0.3700	0.0492	0.0160	3.07 ***
	After matching	0.4192	0.3677	0.0515	0.0180	2.85 ***
Radius	Before matching	0.4192	0.3700	0.0492	0.0160	3.07 ***
	After matching	0.4192	0.3821	0.0371	0.0164	2.27 **
Kernel	Before matching	0.4192	0.3700	0.0492	0.0160	3.07 ***
	After matching	0.4192	0.3747	0.0445	0.0163	2.73 ***

Note: *** and **, respectively indicate that the explanatory variable coefficients are significant at the levels of 1% and 5%.

## Data Availability

The data are available online at https://sites.duke.edu/centerforaging/programs/chinese-longitudinal-healthy-longevity-survey-clhls/data-downloads/ (accessed on 10 June 2018).
